# Endoscopic resection in subepithelial lesions of the upper gastrointestinal tract: Experience at a tertiary referral hospital in The Netherlands

**DOI:** 10.1055/a-2325-3747

**Published:** 2024-07-10

**Authors:** Cynthia Verloop, Lieke Hol, Marco Bruno, Lydi Van Driel, Arjun Dave Koch

**Affiliations:** 1 6993 Department of Gastroenterology and Hepatology, Erasmus Medical Center, Rotterdam, Netherlands; 2 7000 Department of Gastroenterology and Hepatology, Maasstad Hospital, Rotterdam, Netherlands

**Keywords:** Endoscopy Upper GI Tract, Endoscopic resection (ESD, EMRc, ...), Endoscopic ultrasonography, Subepithelial lesions, Tissue diagnosis

## Abstract

**Background and study aims** Histological confirmation of subepithelial lesions (SELs) in the upper gastrointestinal tract remains challenging. Endoscopic resection of SELs is increasingly used for its excellent diagnostic yield and opportunity to do away with continued surveillance. In this study, we aimed to evaluate the indications, success rates and complications of different endoscopic resection techniques for SELs at a large, tertiary referral hospital in Rotterdam, The Netherlands.

**Patients and methods** Data between October 2013 and December 2021 were retrospectively collected and analyzed. Main outcomes were R0-resection rate, en bloc resection rate, recurrence rate, and procedure-related adverse events (AEs) (Clavien-Dindo). Secondary outcomes were procedure time, need for surgical intervention, and clinical impact on patient management.

**Results** A total of 58 patients were referred for endoscopic resection of upper gastrointestinal SELs. The median diameter of lesions was 20 mm (range 7–100 mm). Median follow-up time was 5 months (range 0.4–75.7). Forty-eight procedures (83%) were completed successfully leading to en bloc resection in 85% and R0-resection in 63%. Procedure-related AEs occurred in six patients (13%). Severe complications (CD grade 3a) were seen in three patients. The local recurrence rate for (pre)malignant diagnosis was 2%. Additional surgical intervention was needed in seven patients (15%). A total of 32 patients (67%) could be discharged from further surveillance after endoscopic resection.

**Conclusions** Endoscopic resection is a safe and effective treatment for SELs and offers valuable information about undetermined SELs for which repeated sampling attempts have failed to provide adequate tissue for diagnosis.

## Introduction

Subepithelial lesions (SELs) appear as a mass or bulge covered by normal-appearing mucosa and originate from the gastrointestinal wall 1 . Only 10% to 15% are (pre)malignant and require follow-up or even endoscopic or surgical resection 2 .

Because SELs are covered by normal-appearing mucosa, conventional endoscopy with biopsies is usually insufficient to make a definitive diagnosis. Endoscopic ultrasonography (EUS) is the preferred primary diagnostic modality because of its ability to determine the lesion and the ability to obtain a tissue diagnosis by means of EUS-guided fine-needle aspiration (EUS-FNA) or biopsy (EUS-FNB) 3 4 . The reported diagnostic accuracy for EUS-FNA/B, however, varies widely.

Endoscopic resection can be used as a diagnostic tool and a treatment for small SELs 2 . Different methods for endoscopic resection have been described, including endoscopic mucosal resection, submucosal tunneling endoscopic resection (STER), endoscopic submucosal dissection (ESD), and endoscopic full-thickness resection (EFTR). Compared with surgery, endoscopic resection is less invasive, has short recovery time, and preserves the normal anatomy and function of the digestive tract 5 .

Choice and success rate of the different endoscopic resection methods depends on the layer of origin, lesion location, and the experience of the endoscopist. Rates for complete resection vary between 85% and 98%, with reported adverse event (AE) rates approximately 5% to 10% 5 6 7 . Data about efficacy and complications of endoscopic en bloc resection are limited and mainly derived from Asian countries 8 .

Therefore, this retrospective study aimed to evaluate indications, success rates and complications of different endoscopic resection techniques for SELs in a large academic hospital in The Netherlands.

## Patients and methods

### Patients and data collection

This study was approved by the Medical Research Ethics Committees United (MEC-U). The data analyzed were from patients who underwent endoscopic resections for SELs in the upper gastrointestinal tract at the Erasmus MC Cancer Institute (University Medical Center Rotterdam, The Netherlands) between October 2013 and December 2021. All consecutive adult patients (> 18 years old) who underwent endoscopic resection for a SEL during this time period were included.

The following data were collected: patient demographics, lesion characteristics, such as size (mm), determined by EUS or endoscopy when available, procedure-related outcomes, histological data, and follow-up data.

### Outcome parameters

The main outcomes of this study were technical success rate for endoscopic resection, en bloc resection rate, pathological radical (R0) resection rate, procedure-related AEs, and recurrence rate. Technical success rate was defined as the percentage of procedures in which the intended endoscopic resection technique was completed successfully, without early termination of the procedure or need for conversion to another technique or surgical intervention. En bloc resection rate was defined as number of lesions that were macroscopically complete and removed intact as described by the endoscopist. R0 resection indicated a microscopically margin-negative resection determined by the pathologist. Secondary outcomes were procedure time, need for surgical intervention, and clinical impact of endoscopic resection on patient management. Clinical significance of endoscopic resection was defined as the number of patients who could be discharged from further surveillance following a successfully completed procedure. AEs were graded according to the Clavien-Dindo scoring system 9 .

### Procedures and follow-up

Endoscopic resections described in this study were performed by two experienced endoscopists from the Gastroenterology Department with a colonic full-thickness resection device. The endoscopist determined the type of endoscopic resection based on lesion characteristics such as location, size, and previous imaging.

After all endoscopic procedures, patients were observed in the recovery unit. When there were no signs of delayed complications, patients were discharged on the same day. Patients were prescribed high-dose proton pump inhibitors orally (40 mg twice daily for ≥ 4 weeks) and advised to maintain a clear liquid diet for 12 to 24 hours as per local protocol.

All patients returned to visit the Outpatient Department within 1 to 3 weeks after their procedures for follow-up. Depending on clinical and histopathological outcomes, patients were discharged from further follow-up or entered a surveillance program. When the histopathological diagnosis was gastrointestinal stromal tumor (GIST), NET, or another malignant outcome, the decision about further management was discussed by a multidisciplinary sarcoma or NET team with consideration for lesion features such as size and histopathological risk assessment and patient comorbidities.

## Results

Fifty-eight patients with 67 SELs in the upper gastrointestinal tract were referred for endoscopic resection during the study period and included in this study.

Clinical and patient demographics are shown in Table 1 .

**Table:** **Table 1** Clinical and patient demographics of 58 patients referred for endoscopic resection of upper gastrointestinal SELs.

Demographics	N (%, range)
**Gender, male (n, %)**	27 (47%)
**Age (median, range)**	58 (20–81)
**Incidental findings (n, %)**	27 (47%)
**Indication for endoscopic resection (n, %)**	
Suspected or proven (pre)malignant SEL	34 (59%)
Symptomatic lesion	10 (17%)
Undetermined SEL and patient-preference	12 (21%)
Previous incomplete endoscopic resection	2 (3%)
**Tumor diameter in mm (median, range)**	20 (7–100)
**Tumor location**	
Esophagus	8 (14%)
Stomach	42 (72%)
Duodenum	8 (14%)
**Pre-procedure determined layer of origin**	
Muscularis mucosae	9 (16%)
Submucosa	19 (33%)
Muscularis propria	27 (47%)
Muscularis propria with extraluminal growth	3 (5%)
**Follow-up, months (median, range)**	4,9 (0.4–75.7)
SEL, subepithelial lesion.	

Indications for endoscopic resection were distributed over three major groups: referral because of suspected or proven (pre)malignant diagnosis (e.g. GIST or NET) (n = 34), symptomatic lesion (n = 10) or uncertain diagnosis despite previous attempts for tissue acquisition, and patient reference for lesion removal instead of surveillance (n = 12).

Fifty-eight procedures were initiated, including 22 EFTRs, 28 ESDs, and eight STER procedures. The median diameter of all lesions was 20 mm (range 7–100 mm). Forty-eight of 58 procedures (83%) were successfully completed. En bloc resection was achieved in 41 of 48 patients (85%) and pathological radical (R0) resection in 30 of 48 patients (63%). Procedure-related AEs were seen in seven patients (12%). The overall local recurrence rate after en bloc endoscopic resection was 4% (n = 2) during a median follow-up of 5 months (range 0.4 to 75.7), one inflammatory polyp, and one NET. Additional complementary surgical intervention was needed in seven patients (12%).

Twenty-seven patients (47%) had benign histopathological outcome. Most of them (10 of 27, 37%) were referred for resection because a (pre)malignant diagnosis was suspected, followed by uncertain diagnosis despite previous attempts for histology as reason for resection (9 of 27, 33%). For this reason, benign lesions as heterotopic pancreas were resected.

A total of 31 patients (31 of 58, 53%) had a definitive (pre)malignant histopathological diagnosis (GIST, NET, or leiomyosarcoma) ( Table 2 ). Fifteen of these 31 patients (48.4%) had prior histology with a (pre)malignant histopathological outcome. The other 16 patients were referred because a GIST or NET was suspected based on EUS-features or radiologic imaging. All confirmed GIST lesions were deemed very low to low risk based on mitotic count. Five of eight NETs were radically resected (R0, 62.5%). Of the 19 endoscopically resected GISTs, 10 were R0-resected (52.6%).

**Table:** **Table 2** Overview of histopathological diagnosis of included patients

Histopathological diagnosis	N
Brunner's glands	1
Calcifying fibrous polyp	1
GIST (low-risk)	21
Hamartoma	1
Heterotopic pancreas	7
Inflammatory fibroid polyp	3
Leiomyoma	7
Leiomyosarcoma	2
Lipoma	3
Neuroendocrine tumor	
Low-grade (1)	6
Intermediate-grade (2)	2
Pyloric gland adenoma	1
Reactive cells	1
Unknown because of unsuccessful endoscopic resection	2
GIST, gastrointestinal stromal tumor.	

An overview of the outcomes for endoscopic resection is shown in Table 3 . An overview of procedure-related complications according to Clavien-Dindo classification is shown in Table 4 .

**Table:** **Table 3** Overview of primary and secondary outcomes of endoscopic resection procedures.

	**EFTR**	**ESD**	**STER**
Total number of procedures	22	28	8
Tumor diameter, mm (median, range)	15 (9–25)	21.5 (7–100)	35 (10–60)
Technical success (n, %)	17 (77)	25 (89)	6 (75)
En bloc resection (n, %)	12 (71)	23 (92)	6 (100)
R0-resection (n, %)	13 (76)	13 (52)	4 (67)
R1-resection in premalignant diagnosis (n, %)	3 (18)	8 (29)	1 (17)
Local recurrence (n, %)	1 (6)	1 (4)	–
Procedure time, min (median, range)	35.5 (19–120)	72 (9–240)	138.5 (44–487)
Additional surgical intervention (n, %)	2 (9)	5 (18)	–
Post-procedure complications (n, %)	2 (9)	4 (14)	1 (13)
EFTR, endoscopicfull-thickness resection; ESD, endoscopic submucosal dissection; STER, submucosal tunneling endoscopic resection.			

**Table:** **Table 4** Overview of procedure-related complications.

Clavien-Dindo classification	EFTR	ESD	STER	Total
No complications (n, %)	20 (91)	24 (86)	7 (88)	51 (88)
1 (n, %)	1 (5) Post-ERCP pancreatitis	2 (7) Nausea and pain	–	3 (5)
2 (n, %)	–	–	–	–
3 (n, %)	1 (5) – Obstruction of common bile duct due to OTS-clip	2 (7) Additional endoscopy for bleeding	1 (13) Additional endoscopy for pain after dehiscence of mucosal access	4 (7)
4 (n, %)	–	–	–	–
5 (n, %)	–	–	–	–
EFTR, endoscopic full-thickness resection; ESD, endoscopic submucosal dissection; STER, submucosal tunneling endoscopic resection; OTS, over-the scope; ERCP, endoscopic retrograde cholangiopancreatography.				

### EFTR

EFTR was successful in 17 of 22 patients (77%) with a median lesion size of 15 mm (range 9–25). Most EFTR were performed in the stomach (n = 14), the other eight were performed in the duodenum. Endoscopic en bloc resection was accomplished in 12 of 17 procedures (71%) ( **Supplementary Fig. 1** ). Three of the lesions (18%) that were not radically resected (R1) concerned a NET (n = 3) (grade 1–2), but none recurred during a median follow-up time of 8 months (range 0.3–75.7).

In five of 22 patients, EFTR was unsuccessful because the lesion could not be pulled into the cap because of size or rigidity. Eventually, two of five patients with incomplete procedures were referred for surgical wedge excision of the lesions because there was a high suspicion of a malignant diagnosis, two of five patients were rescheduled for ESD leading to successful resection of GIST, and one patient continues to be monitored (47 months follow-up).

Two patients (9%) experienced procedure-related complications. In both cases, the (pre)malignant lesions were located in the duodenum, near the ampulla of Vater. Given patient comorbidities, surgical intervention was not viable and endoscopic resection was deemed to be the second-best option. One patient developed a mild post-ERCP pancreatitis after pre-procedure placement of a protective stent in the common bile duct (Clavien-Dindo 1). Another patient developed obstruction of the common bile duct due to the position of the OTS-clip. Later, the OTCS-clip was removed. (Clavien-Dindo 3a).

### ESD

Of 28 initiated ESD-procedures, 25 (89%) were successful ( **Supplementary Fig. 2** ) in lesions with a median size of 21.5 mm (range 7–100). All ESDs were performed in the stomach. Three procedures were unsuccessful due to extraluminal growth (n = 2) or because the lesion was too large to resect endoscopically (n = 1). The en bloc resection rate was 92% (23/25) and the R0 resection rate was 52% (13/25). In patients with R1-resection, eight of 12 lesions (29%) were low-risk GIST and the other lesions were heterotopic pancreas (n = 3 of 12) and an inflammatory polyp (n = 1 of 12). Despite R1-resection of GIST, five of eight patients were discharged because of benign characteristics and en bloc resection of the lesion. Local recurrence after ESD developed in one patient during a median follow-up time of 7 months (range 0.4–69.3), this was a grade I NET.

In one frail elderly patient, a symptomatic GIST was too large (60 mm) to be removed after ESD through the mouth. Consequently, the tumor was left in the stomach following dissection, making assessment of resection margins unfeasible. Resection margins, therefore, could not be evaluated. Surgical intervention was not considered an option for the patient. A follow-up visit occurred 2 months after the intervention. The symptoms subsided and the patient remained in good health. Therefore, further monitoring was deemed unnecessary.

As shown in Table 4 , four patients experienced post-procedure complications. Two patients presented with melena and needed additional endoscopy to treat post-procedure bleeding (Clavien-Dindo 3a). Two patients needed hospitalization for observation of symptoms such as nausea or pain (Clavien-Dindo 1).

Additional surgery (n = 5) was successfully performed in four patients with an unsuccessful endoscopic resection, resulting in radical resection of three GISTs and one leiomyoma, and in one patient with an R1- resection of a GIST.

### STER

All patients underwent STER because the lesion was located in the esophagus. The lesions had a median size of 35 mm (range 10–60 mm). Of eight procedures, six were successful (75%) and en bloc resection was achieved ( **Supplementary Fig. 3** ). Despite circumferential dissection, one lesion could not be removed because of fibrosis. The supplying blood vessels were transected and the lesion was left in situ. Per-procedural biopsies showed leiomyoma. After 3 months, the lesion decreased in size and the patient reported resolution of dysphagia.

Lesions with irradical resection margins were all leiomyomas. However, radical resection could not be determined in one patient due to lesion damage when it passed through the upper esophageal sphincter. This was a low-grade leiomyosarcoma for which surveillance was advised in the referring hospital.

One patient was hospitalized after the procedure because of progressive thoracic pain caused by a post-procedure dehiscence of the mucosal access with spill into the submucosal tunnel. The dehiscence was closed by clipping a mucosal flap over the defect. The patient recovered quickly with additional antibiotics (Clavien-Dindo 3a).

### Clinical impact on patient management

**Fig. 1:**
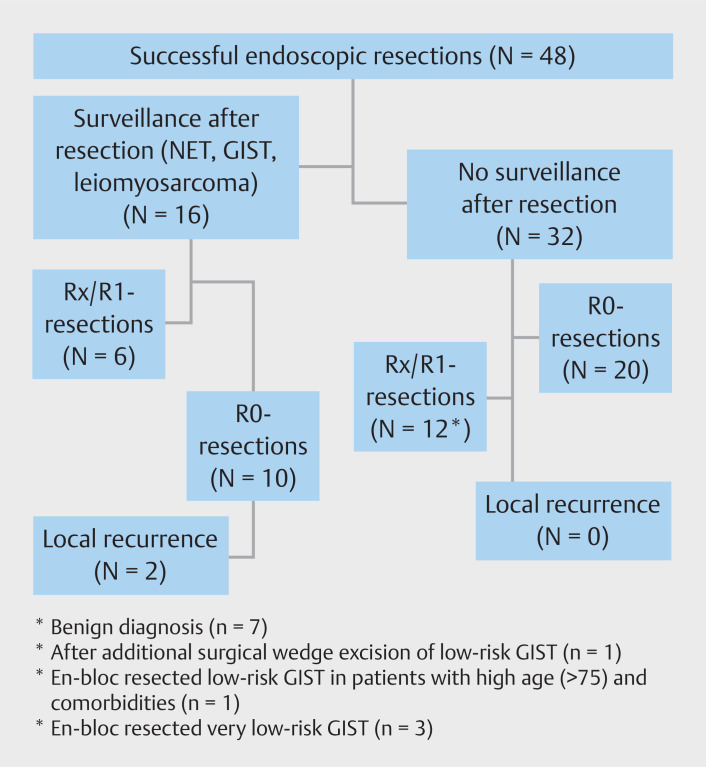
Clinical impact of endoscopic resections. GIST, gastrointestinal stromal tumor; NET, neuroendocrine tumor; R0, pathological radical resection; R1, pathological irradical resection.

An overview of the clinical impact of the endoscopic resections in this study is shown in Fig. 1 .

After 48 completed procedures, 32 patients (67%) were discharged from further surveillance post-procedure. The majority of these patients (20 of 32) had R0-resection of a histopathological benign lesion (n = 13) or low-risk (pre)malignant diagnosis (n = 7). In addition, 12 patients with R1-resections could also be discharged from further follow-up because of benign histopathological outcome (n = 7), a diagnosis of low-risk GIST in a patient with severe comorbidities (n = 1), or benign characteristics in the histopathological sample in combination with en bloc resection of the lesion (n = 3). In one discharged patient with R1-resection of a low-risk GIST, an additional surgical wedge excision was achieved, which showed no residual malignant cells in the histopathological sample. No local recurrence is known to have occurred in these patients during a median follow-up time of 1 month (range 0.3–69.3).

Sixteen patients (33%) entered a surveillance program. Three patients had irradical resection margins of GISTs and three other patients had low-grade NETs. The other 10 patients had radical resection margins, but the histopathological diagnosis (e.g. NET [n = 6], large, recurrent or multiple GIST [n = 3] or leiomyosarcoma [n = 1]) necessitated that the patient enter a surveillance program. During a median follow-up period of 7 months (range 0.4–69.3), one patient developed local recurrence of a NET after undergoing ESD and one patient had local recurrence of a symptomatic inflammatory fibroid polyp after EFTR.

## Discussion

Adequate tissue sampling is essential to achieve a diagnosis that distinguishes between (pre)malignant lesions requiring follow-up or resection and non-neoplastic lesions, which require no additional surveillance. In addition, histological diagnosis is important for risk stratification and subsequent management of a NET and a GIST. Current tissue acquisition methods, however, have their limitations and the optimal management strategy remains unclear, especially for small SELs 10 . This retrospective clinical data study in 58 patients with SELs demonstrated that endoscopic resection could be a safe and effective treatment with a technical success rate of 83% and AE rate of 12%, achieving en bloc resection and R0-resection in 85% and 63%, respectively. In addition, in only 12%, additional surgery was needed and 67% of patients could subsequently be discharged from further surveillance after successful endoscopic resection.

Guidelines suggest obtaining tissue with EUS-FNA, EUS-FNB, or mucosal incision-assisted biopsy (MIAB) 2 10 11 . In clinical practice, EUS-FNA and EUS-FNB are the most widely used but have a poor diagnostic yield especially in lesions < 20 mm 12 13 14 . Current literature suggests that MIAB techniques result in higher diagnostic yield 15 16 17 . However, the reported diagnostic yield of these techniques is limited in small lesions (< 20 mm) (47% to 79%) 12 13 14 and all available MIAB techniques can result in local fibrosis, which may hamper future attempts at endoscopic resection using, for instance, submucosal tunneling 18 19 20 . This study indicates that endoscopic resection can be considered an effective diagnostic tool for small SELs with 36 of 58 SELS (62%) being < 20 mm (range 7–100) 15 16 21 .

Undiagnosed SELs often require intensive surveillance 2 10 , which may lead to a significant burden especially in young patients. In addition, previous data showed a low compliance (44.6%) for the recommended surveillance strategy 22 . In the most recent European Society of Gastrointestinal Endoscpy guideline for management of SELs, it is therefore suggested that resection is an option for undetermined SELs of < 20 mm to avoid the need for intensive follow-up 10 .

The challenge remains in setting correct eligibility criteria for choosing endoscopic resection. Because the current study was performed with clinical data from a tertiary referral hospital specialized in GIST and NET treatment, most patients (59%) were referred for endoscopic resection because of a high suspicion or proven (pre)malignant SEL. Twelve patients (21%) were referred for endoscopic resection because of an undetermined SEL for which the patient expressed a preference to resection instead of surveillance. In this group with a median diameter of SEL of 18.5 mm (range 9–30 mm), the technical success rate was 91.7% (11 of 12 procedures) with a pathological (R0) resection rate of 54.5% (6 of 11 procedures). Long-term surveillance could be prevented in eight of 11 patients because of benign histopathological outcome (n = 6) or R0-resection of low-risk premalignant lesion (n = 2). These results suggest that even when a R0-resection is not achieved, endoscopic resection can be safe and effective in managing small, undetermined SELs, because the majority are benign.

When considering the clinical impact of successful endoscopic resection, this study showed that of 48 patients undergoing successful endoscopic resections, 32 (67%) could be discharged from follow-up. There are no direct comparisons between a follow-up strategy and direct diagnostic excision strategy. However, the findings in this study might indicate that endoscopic treatment can contribute to preventing a patient from unnecessary diagnostic and therapeutic procedures through diagnosis of an undetermined lesion or cure of a malignant lesion.

Surgical wedge excision is considered to be the gold standard in Western guidelines for treatment of (malignant) SELs. In agreement with previous studies, the choice for an endoscopic resection technique was dependent on lesion diameter and location, and local expertise 7 23 . There are no previous studies directly comparing the different endoscopic resection techniques. In the current study, AEs were seen in 12% (7/58 procedures), but these were only severe (CD ≥ 3) in 7% (4 of 58 procedures) and could be quickly resolved. These rates are consistent with previously reported AE rates, which range from 5% to 15% 7 24 . The AE rates for endoscopic resection are comparable to those for laparoscopic resection techniques 25 26 . Endoscopic resection, therefore, can be considered a safe, less invasive alternative for both diagnosing and treating SELs, with shorter procedure time, less blood loss, and shorter hospital stay 25 27 .

The optimal treatment of small GISTs still remains controversial. For intraluminal GISTs smaller than 20 mm, resection and surveillance are both acceptable alternatives. For lesions up to 35 mm, endoscopic resection may be an alternative to laparoscopic wedge excision 10 28 . The current study shows high technical success rates for EFTR (77%), ESD (89%), and STER (75%), en bloc resection rates of 71%, 92%, and 100%, respectively. These outcomes for ESD are in accord with previous literature, but higher successful resection rates are reported for EFTR and STER 7 29 30 . A possible explanation for this could be that the average diameter of resected lesions in this study was relatively large, which might have hampered successful performance of EFTR and STER. However, subgroup analysis demonstrated only an improved success rate for STER in lesions with diameter ≤ 30 mm (n = 4; 100%). Good visibility and the ability to successfully resect the lesion in EFTR and STER is limited by the maximum diameter of the cap size for EFTR and the upper esophageal sphincter for STER. Also, some SELs are fixed to the surrounding gastrointestinal wall, making it difficult to capture in the cap. Reported complete resection rates in literature range from 74% to 100%, with higher rates reported for lesions originating from the third wall layer and of smaller size 31 32 . In ESD, the resection margin is close-fitted to the SEL and evaluation of the pathological margin is more difficult, which might explain the lower reported pathological resection rate of 52% found in the current study. However, no local recurrence was seen in the R1-resected lesions. The local recurrence rate for premalignant diagnosis in this study was only 2% during follow-up. In addition, the implication is that R1 resection in GIST is not associated with a higher risk of local recurrence or lower survival outcome as long as en bloc resection is achieved 33 .

Although the present results support the feasibility and effectiveness of endoscopic treatment of SELs, it is appropriate to recognize several limitations of the study. It was a retrospective evaluation of clinical data from an experienced referral tertiary center. Important data or nuances could be missed when the information was not documented in electronic health records. Even though most patients were discharged from further surveillance, the follow-up time in the remaining patients was modest. The possibility of long-term recurrence, therefore, cannot be completely ruled out. In addition, because the procedures were performed by experienced endoscopists in a tertiary referral center, data cannot be transposed to smaller, regional centers with less experience for this specific indication.

## Conclusions

In conclusion, endoscopic resection is an effective treatment for SELs and offers valuable information about undetermined SEL in a field hampered by low diagnostic accuracy of current techniques. In addition, in the current study, two-thirds of the referred patients could be discharged from surveillance and unnecessary follow-up procedures were prevented. Eligibility criteria and the long-term recurrence rate for endoscopic resection are not yet well-established and need further investigation.

## Supplementary Material

Supplementary Material Zusatzmaterial
