# Supplementary material for: Endoscopic resection in subepithelial lesions of the upper gastrointestinal tract: Experience at a tertiary referral hospital in The Netherlands
Source: Endosc Int Open. 2024 Jul 10;12(7):E868–74. doi: 10.1055/a-2325-3747 (PMC11236476; doi:10.1055/a-2325-3747)

Supplementary material

Supplementary Fig. 1 EFTR-procedure for a gastric neuroendocrine tumor. **a** Marked nodule lesser curvature. **b** Resection surface above the above the deployed over-the-scope clip (OTSC). **c** Full-thickness resection of the lesion pinned down on foamboard.

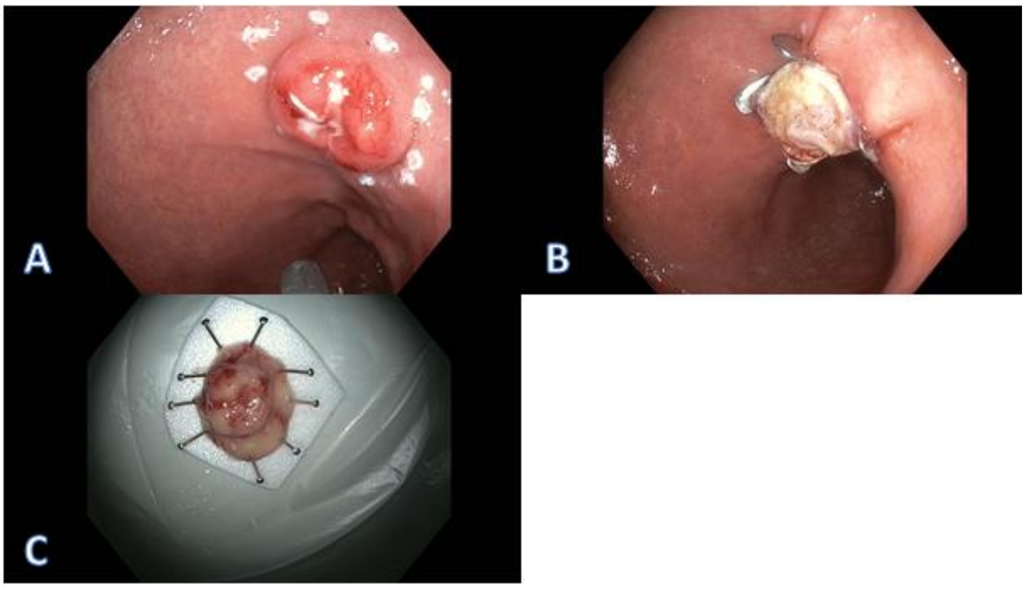

Supplementary Fig. 2 ESD procedure for a gastrointestinal stromal tumor. **a** Gastric subepithelial lesion. **b** Submucosal dissection of the lesion. **c** Resection surface.
d View of en bloc resection lesion.

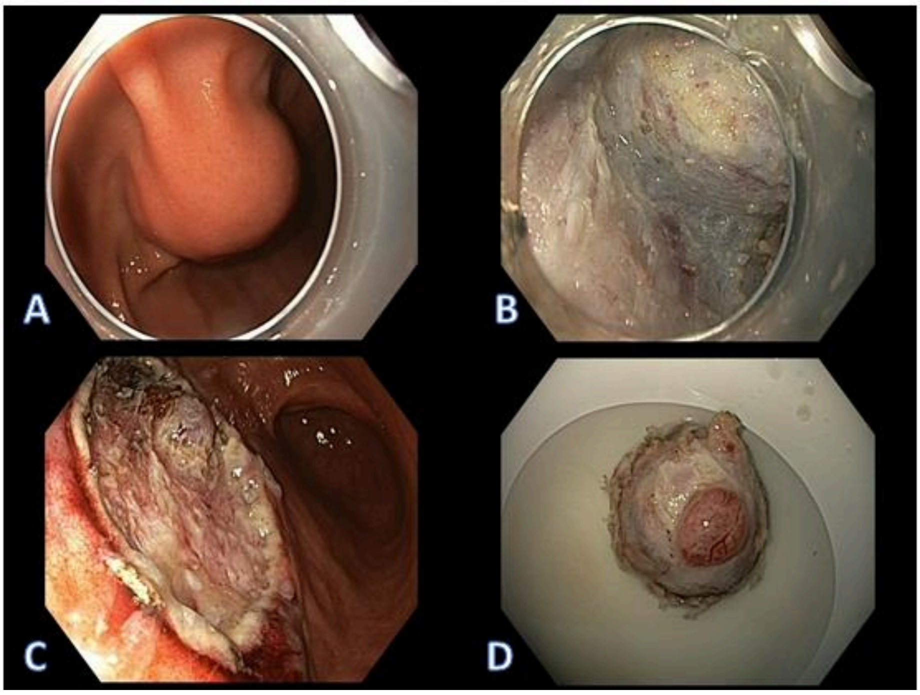

Supplementary material

Supplementary Fig. 3 STER-procedure of a leiomyoma. **a** View of subepithelial lesion in esophagus. **b** Submucosal tunneling. **c** Dissection of lesion out of tunnel. **d** View of tunnel before closing the entry.

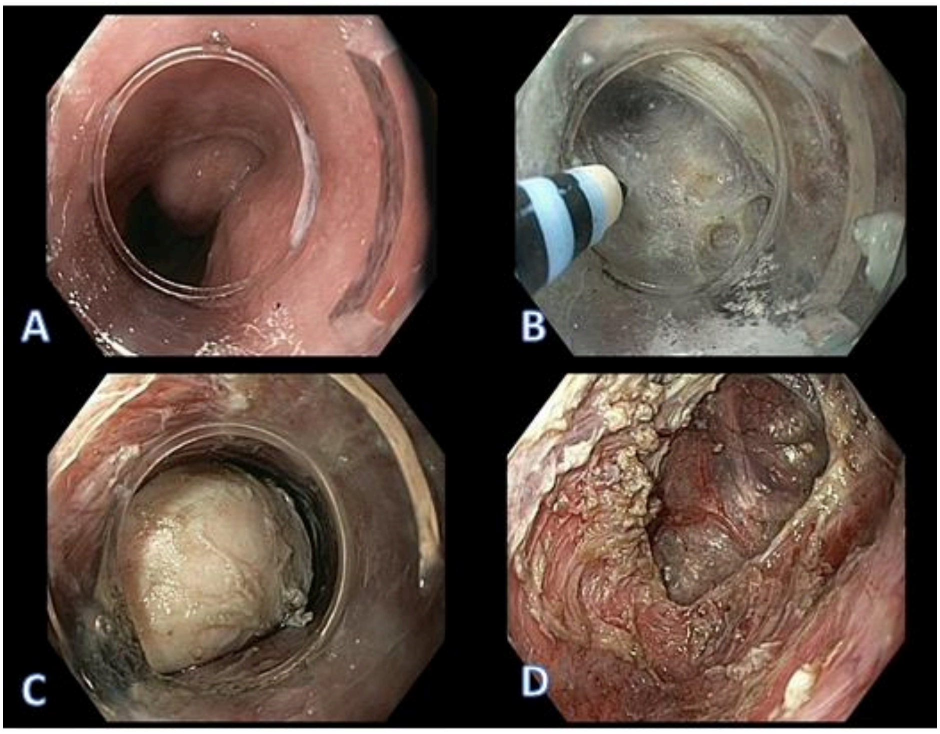

Supplement: Supplementary file 1 — Supplementary Material [file 10-1055-a-2325-3747_23318976.pdf]
